# Seasonal host dynamics drive the timing of recurrent epidemics in a wildlife population

**DOI:** 10.1098/rspb.2008.1732

**Published:** 2009-01-20

**Authors:** Michael Begon, Sandra Telfer, Matthew J. Smith, Sarah Burthe, Steve Paterson, Xavier Lambin

**Affiliations:** 1 School of Biological Sciences, The University of LiverpoolLiverpool L69 7ZB, UK; 2 Computational Ecology and Environmental Science Group, Microsoft Research Ltd.Cambridge CB3 0FB, UK; 3 Institute of Biological and Environmental Sciences, University of AberdeenAberdeen AB24 2TZ, UK

**Keywords:** epidemic, cowpox virus, field vole, wildlife, seasonal, infection

## Abstract

The seasonality of recurrent epidemics has been largely neglected, especially where patterns are not driven by forces external to the population. Here, we use data on cowpox virus in field voles to explore the seasonal patterns in wildlife (variable abundance) populations and compare these with patterns previously found in humans. Timing in our system was associated with both the number and the rate of recruitment of susceptible hosts. A plentiful and sustained supply of susceptible hosts throughout the summer gave rise to a steady rise in infected hosts and a late peak. A meagre supply more limited in time was often insufficient to sustain an increase in infected hosts, leading to an early peak followed by a decline. These seasonal patterns differed from those found in humans, but the underlying association found between the timing and the supply of susceptible hosts was similar to that in humans. We also combine our data with a model to explore these differences between humans and wildlife. Model results emphasize the importance of the interplay between seasonal infection and recruitment and suggest that our empirical patterns have a relevance extending beyond our own system.

## 1. Introduction

To understand many infectious disease systems, it will become increasingly important to understand the multiple and often neglected effects of seasonality (Altizer *et al*. 2006). Acknowledging that seasonal patterns themselves are highly labile in the face of recent climate change (Walther *et al*. 2002) adds further weight to this. It is apparent from Altizer *et al*.'s (2006) review that one particularly neglected topic is variation, within a system, in the size and timing of seasonal outbreaks. Stone *et al*. (2007) however, have addressed this question in human infections. Using mathematical models supported by data on measles and mumps, they found that the size of epidemic peaks (in years when there was an epidemic peak) tended to be smaller when epidemics occurred later in the year, but that these late-phase epidemics tended to precede larger epidemics the following year. Late-phase epidemics were often so small that the year could be described as a ‘skip’: no epidemic having occurred. Whether a year was a skip year depended in their model on a threshold, determined by the number of susceptible hosts remaining after 1 year's epidemic and the rate of recruitment of new susceptible individuals into the population during the following year.

Stone *et al*. (2007) pointed out however, that further work was required to extend the analysis to cases in which the population size and the birth rate varied. This would be true, for example, moving beyond the relatively simple medical context to infections in wildlife populations, which may be of interest either for their potential role in wildlife dynamics (Hudson *et al*. 2002) or because of the zoonotic threat they pose (Taylor *et al*. 2001). Mostly, the large datasets available in the medical literature have no counterparts for wildlife infections. Here though, we are able to use data on cowpox virus infection in populations of the field vole, *Microtus agrestis*, to seek patterns comparable to those found by Stone *et al*. (2007). We also combine these data and a model pertinent to our populations (Smith *et al*. 2008) to begin to explore how and why seasonal patterns of infection may differ between human and wildlife populations.

For continuity, we retain the word ‘epidemic’ to describe an annual peak in the dynamics of cowpox virus infection, without requiring that this should substantially exceed endemic levels. However, we refer to the annual ‘timing’ rather than the ‘phase’ of epidemics, since our host populations undergo multi-annual cycles (Lambin *et al*. 2000), which are, by convention, divided into ‘peak’, ‘crash’ and other phases. We also note that Stone *et al*. (2007) studied both the numbers infected and the prevalence of infection (the proportion of the population that is infected), because the two are interchangeable in populations of constant size. Here, therefore, where the two have distinct dynamics because population size varies, we also study both. Specifically, we first examine the neglected relationships (Altizer *et al*. 2006) between the timing and the size of current and subsequent epidemics. We also investigate, directly, the more general proposition that the timing of an epidemic is related to the recruitment of susceptible hosts into the population. Furthermore, since, unlike in human infections, host abundance here is itself dynamic, we examine the relationship between the timing of epidemics and a key aspect of host dynamics, the phase of the multi-annual cycle.

Finally, we study the predictions of the Smith *et al*. (2008) model, shown previously to recreate key features of our study system, and initiate a comparison of the predictions of the model with our empirically derived relationships. The model assumes seasonal reproduction and a constant infection rate, in contrast to Stone *et al*. (2007), although the latter uses a seasonally varying contact rate. Hence, we ask whether these differing assumptions may lead to differing predictions and explain any differences between our empirical relationships and the predictions of Stone *et al*. (2007).

## 2. Material and methods

### (a) Study area and trapping design

The study took place in Kielder Forest, a man-made spruce forest occupying 620 km^2^, situated on the English-Scottish border (55°13′ N, 2°33′ W). Field voles inhabit grassy clear-cuts that represent 16–17% of the total area, but are completely absent from forested areas that isolate the clear-cuts. Clear-cuts range in size from 5 to 100 ha. Field vole populations at Kielder fluctuate cyclically with a 3–4 year period (Lambin *et al*. 2000). Biannual estimates (spring (March) and autumn (September–October)) of the population density (in voles per hectare) of field voles were undertaken from summer 1984 to spring 2008 in 16–21 grass-dominated clear-cut areas or unplanted river meadows in Kielder Forest, so as to derive a landscape scale estimate of vole cycle phase. Density estimation is based on a calibrated index (the ‘vole signs index’, VSI) scoring the presence/absence of feeding signs (unoxidized grass clippings) in 25 25×25 cm quadrats at each site. A full description of the method of data collection and the abundance estimation procedure can be found in Lambin *et al*. (2000).

Voles were trapped in four similar-sized clear-cuts, in two areas of the forest approximately 12 km apart, between May 2001 and March 2007. In the Kielder catchment, Kielder Site (KCS) and Plashett's Jetty (PLJ) are situated 4 km apart. In the Redesdale catchment, Black Blake Hope (BHP) and Rob's Wood (ROB) are 3.5 km apart. These four populations were far enough apart, with sufficient forest between them, to be considered as effectively independent replicates.

Populations were trapped over 3 days every 28 days from March to November, and every 56 days from November to March. Each site had a permanent 0.3 ha live-trapping grid consisting of 100 Ugglan Special Mousetraps (Grahnab, Marieholm, Sweden) set at 5 m intervals. Individual animals were identified using subcutaneous microchip transponders (AVID plc, East Sussex, UK) injected into the skin at the back of the neck. A 20–30 μl blood sample was also taken from the tail tip of each individual each trapping session. Antibody to cowpox virus was detected in sera by immunofluorescence assay (Crouch *et al*. 1995), allowing individuals in each primary session to be classified as seropositive (antibody present) or seronegative. For further details see Begon *et al*. (in press).

### (b) Cowpox virus data

Animals infected with cowpox virus develop an antibody response after approximately two weeks, and remain infected for a period of approximately four weeks, following which they recover but remain seropositive (Bennett *et al*. 1997; Chantrey 1999; Blasdell 2006). Therefore, in a time series of antibody results, we assumed that an animal became infected with uniform probability between a time 14 days prior to its last negative result, and 14 days prior to its first positive result. Time series of serological results were thus used to calculate probabilities that individual animals were infected with cowpox virus (*p*(*I*)), were still susceptible (*p*(*S*)) or had recovered and were resistant (*p*(*R*)) for each trapping session. These were used to subdivide the total population into *I*, *S* and *R* individuals, that total itself being estimated in program Mark using Huggins's closed capture model within a robust design (Huggins 1989; Kendall & Nichols 1997; Pledger 2000). Telfer *et al*. (2002) and Begon *et al*. (in press) have provided detailed descriptions of the calculation of probabilities, but to take a simple example: an individual caught negative at trap sessions *t*−2 and *t*−1, and positive at *t* and *t*+1 (all four weeks apart) must have become infected during the period from two weeks prior to trap session *t*−1 until two weeks prior to session *t* (see above). Hence, it would have a 0.5 probability of being infected at *t*−1 (*p*(*I*)=0.5) and a 0.5 probability of being infected at *t*. It must still have been susceptible at session *t*−2 (*p*(*S*)=1) and must have recovered and acquired resistance at session *t*+1 (*p*(*R*)=1). At session *t*−1, if not infected, it must still have been susceptible (*p*(*S*)=0.5); and at session *t* it must have been resistant if not infected (*p*(*R*)=0.5). Individual *p*(*I*), *p*(*S*) and *p*(*R*) values, calculated in this way, can then be summed up for each trap session to estimate the proportion in the sample that are infected, susceptible and resistant. The total abundance, *N*_*t*_, estimated as described above, may then be subdivided into its components, *I*_*t*_, *S*_*t*_ and *R*_*t*_ on the basis of these proportions.

### (c) Data analysis

Analyses were carried out on the natural logarithms of abundances (total, infected and susceptible), as these translate multiplicative changes (stemming from *per capita* vital rates) into additive ones (Turchin 2003). As previously noted, epidemics were investigated using both ln(*I*) and the prevalence of infection. Associations were sought between the timing of peaks (a peak was the single highest lunar month, 1–13, classed as an ordered factor) and their size (both current and subsequent) through generalized linear models (binomial in the case of prevalence) in the statistical package R (The R Foundation for Statistical Computing; http://www.r-project.org/). To test the hypothesis that the timing of epidemic peaks was related to the input of susceptible hosts, timing was the response variable and hence ordinal regression was applied, using the function *lrm* in the design package in R. The predictor variables were the rate of increase in the number susceptible over the summer concerned, i.e. ln(number susceptible in month 10/number susceptible in month 3) and the sum of the number of susceptible hosts present each month from months 3 to 10 inclusive. This and other demographic characters change through the phases of the host's multi-annual abundance cycle (Ergon *et al*. 2001). Hence, ordinal regression was again applied to test whether any relationships between the timing of epidemic peaks and numbers susceptible were related in turn to the phase of the host abundance cycle, this time with peak ln(*N*) during the previous summer as the predictor variable (the highest in the peak phase and the lowest in the crash phase).

### (d) The rodent–pathogen dynamics model

In the model of Smith *et al*. (2008), the host population density is divided into four classes of individuals: those that are susceptible to microparasitic infection, *S*; infected individuals that cannot reproduce, *I*; recovered and immune individuals that cannot yet reproduce, *Y;* and recovered and immune individuals that can reproduce, *Z*. The change in the densities of the host classes over continuous time, *t*, is given by

(2.2a) $$\frac{\text{d}S}{\text{d}t}=A(t)(S+fZ)(1-qN)-\beta SI-bS,$$

(2.2b) $$\frac{\text{d}I}{\text{d}t}=\beta SI-(b+\alpha +\gamma )I,$$

(2.2c) $$\frac{\text{d}Y}{\text{d}t}=\gamma I-(b+\tau )Y,$$

(2.2d) $$\frac{\text{d}Z}{\text{d}t}=\tau Y-bZ,$$

where

$$A(t)=\{\begin{array}{cc} a & T<t<T+L,\\ 0 & T+L<t<T+1. \end{array}$$

Here, *L* is the reproductive season length in units of a fraction of 1 year, *T* is the time in integer years and *N*=*S*+*I*+*Y*+*Z* is the total population density. The disease-free *per capita* death rate, *b*, is constant throughout the year but the *per capita* birth rate is seasonal, (*A*(*t*)), with no births possible in the non-reproductive season (*A*=0) and a constant maximum *per capita* birth rate in the reproductive season (*A*=*a*). The birth rate is assumed to be density dependent and is modified owing to a crowding coefficient*, q*, which is related to the carrying capacity, *K*=(*a*−*b*)/*aq*. There is density-dependent transmission at rate *β*. Infected individuals potentially have an increased mortality rate owing to the effects of the disease (*α*), and recover at a constant rate *γ*. Recovered individuals initially enter an immune but non-reproductive class which they leave at a rate *τ* and regain a proportion of their reproductive capacity *f* (0<*f*<1).

Smith *et al*. (2008) have parametrized this model for a number of rodent populations including field voles in Kielder Forest. Here, we use the parameter values for the Kielder Forest field voles with disease parameter values, plausible for cowpox virus, that lead to irregular cycles with a dominant multi-year periodicity of 4 years. We base our analysis on the long-term dynamics predicted by the model (visible transients having disappeared after 50 years, we sampled 100 years of data for analysis, starting at year 500). To enhance comparability with the field data, we added stochasticity to the model by randomly perturbing the maximum birth rate parameter by a normally distributed amount with a mean of zero and a standard deviation of 0.5. This occurred at the start of each reproductive season and subsequently remained constant for its duration. For comparability with the analyses of field data described above, and because the timing of peaks in prevalence and numbers infected were almost perfectly correlated (§3), relationships were examined between the size and timing of peak prevalence, and the timing of peak prevalence and both the rate of increase in *S* over the breeding season and *N* at the end of the previous breeding season (when *N* peaks).

## 3. Results

**Figure 1:**
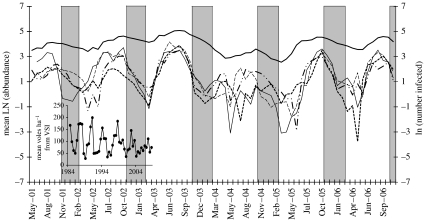
The dynamics of the natural log of estimated abundance (the mean for the four sites, each 0.3 ha: ‘mean LN’ (thick solid curve)) and of the natural logs of the estimated numbers infected at the four sites: BHP (log number of BHP infecteds, long dashed curve); KCS (log number of KCS infecteds, dot dashed curve); PLJ (log number of PLJ infecteds, short dashed curve); and ROB (log number of ROB infecteds, thin solid curve). The inset shows biannual estimates of the population density (in voles per hectare) from summer 1984 to spring 2008 in 16–21 sites in Kielder Forest, using the VSI.

Figure 1 shows the dynamics of infection at the four sites (the estimated numbers infected in the populations at the time of each sample) along with the overall dynamics of the host over the same period: the estimated total abundance at each sample point averaged over the four sites for clarity. These natural dynamics of abundance are clearly very different from the common assumption of a constant population size in epidemiological models of human populations. The field vole population displayed a clear annual cycle of abundance, peaking in late summer or autumn each year and falling to a trough in spring or early summer. Multi-year changes in the population size are also apparent, rising to a peak in 2003 and falling to a trough in 2004. VSI dynamics before, during and after the sampling period are shown as an inset and reveal that these population dynamics are part of a series of multi-year cycles, with the peak phase in 2003 as well as the subsequent peak in 2007 and crash in 2008, after the completion of this study. In addition, details of the annual cycle varied over the course of the multi-annual cycle. The earliest annual maximum abundance occurred in the summer of the peak year of the multi-annual cycle, and the latest annual minimum was in trough phase of the multi-annual cycle.

Contrary to the findings of Stone *et al*. (2007), the sizes of the peaks in the number of infected individuals were not associated with the timing of those peaks in either the same year (*z*_24_=0.81, *p*=0.43) or the previous year (*z*_24_=0.56, *p*=0.59). Similarly, the sizes of the peaks in prevalence were not associated with the timing of those peaks in the same year (*z*_24_=0.39, *p*=0.76) or the previous year (*z*_24_=0.12, *p*=0.91). The timings of the peaks in prevalence and numbers infected were themselves strongly associated, with the exception of one site (ROB 2004), which peaked for numbers infected in month 3 and for prevalence in month 10.

**Figure 2:**
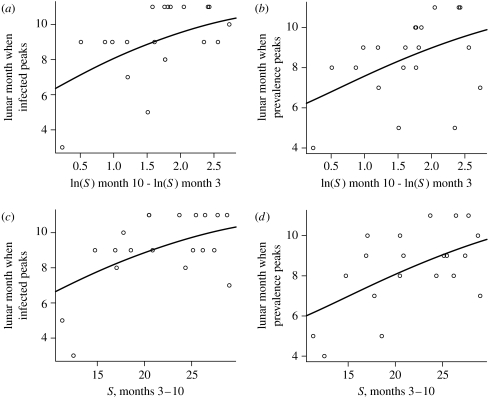
Ordinal regressions for the relationships between (*a*) the lunar month when the numbers infected peak and the rate of increase in the number susceptible over the summer concerned, i.e. ln(number susceptible in month 10)−ln(number susceptible in month 3), (*b*) the lunar month when prevalence peaks and the rate of increase in the number susceptible, (*c*) the lunar month when the numbers infected peak and the overall number susceptible present over the summer concerned, and (*d*) the lunar month when prevalence peaks and the overall number susceptible. Statistics are given in table 1.

The timing of epidemic peaks, however, was strongly associated with the dynamics of susceptible hosts. Epidemic peaks occurred significantly later when the rate of recruitment of susceptible hosts over the summer (March–September) was greater (figure 2*a*,*b*), whether these were peaks in the numbers infected (table 1, model N1) or in prevalence (table 1, model P1). Peaks occurred significantly later, too, when the sum of the number of susceptible hosts present over these summer months was greater (figure 2*c*,*d*), again whether these were peaks in the numbers infected (table 1, model N2) or in prevalence (table 1, model P2). In fact, the effects of these two predictor variables appear to be additive, since ordinal regressions that included both explained significantly more of the variation (table 1, model N3 compared to models N1 and N2, and model P3 compared to models P1 and P2). Furthermore, an interaction term between the two variables was non-significant, and the coefficients for the individual variables were essentially unchanged when both were included (table 1).

**Figure 3:**
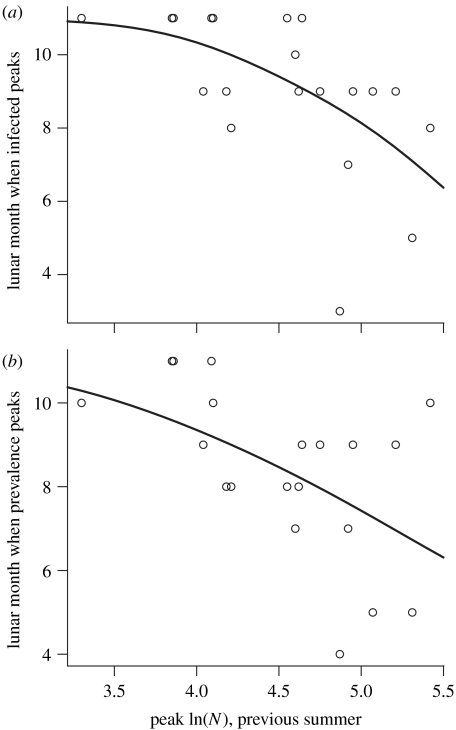
Ordinal regressions for the relationships between (*a*) the lunar month when the numbers infected peak and the peak ln(abundance) during the previous summer; and (*b*) the lunar month when prevalence peaks and the peak ln(abundance) during the previous summer. Statistics are given in table 1.

These patterns relating timing to host dynamics were related, in turn, to the phase of the multi-annual cycle (figure 3*a*,*b*). Epidemic peaks occurred significantly later following summers of lower maximum abundance (i.e. tended to be the latest in the summer of an ‘increase’ year), whether these were peaks in the numbers infected (table 1, model N4) or in prevalence (table 1, model P4). In fact, cycle phase appeared to be a proxy for the number (rather than the rate of recruitment) of susceptible hosts, since ordinal regressions with both peak ln(*N*) in the previous year and rate of recruitment explained significantly more of the variation than either did alone (table 1, model N5 compared to models N1 and N4, and model P5 compared to models P1 and P4), whereas adding the number of recruits to the ordinal regression with peak ln(*N*) did not improve explanatory power (table 1, model N6 compared to models N2 and N4, and model P6 compared to models P2 and P4).

**Figure 4:**
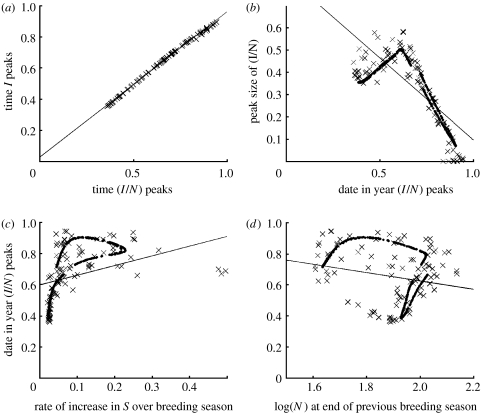
Predictions of the seasonal host–microparasite model. Black dots are the predictions from the deterministic (noise-free) model and crosses are those from the model with a stochastic birth rate (see text). Lines of best fit were estimated by linear regression, using the data from the stochastic model. (*a*) The time of the year in which prevalence (*I*/*N*) peaks is always tightly correlated with the time at which the numbers infected (*I*) peaks. The deterministic data are omitted but also lie on the best-fit line. (*b*) Peak prevalence appears to be negatively related to the date at which it peaks, although the deterministic attractor suggests a humped relationship. (*c*) The rate of increase of the number susceptible, *S*, over the breeding season is generally positively related to the date at which prevalence peaks. (*d*) The general relationship between the date at which prevalence peaks and abundance, *N*, at the end of the previous breeding season is slightly negative although the deterministic attractor shows a highly nonlinear relationship.

Patterns with similarities to those observed in the data were also apparent in the model output, for both the numbers infected and the prevalence, though in view of the tight correlation between them (figure 4*a*; linear regression *R*^2^=0.997) only the latter are presented. Thus, as the timing of the epidemic peak gets later, the size of that peak initially increases slightly (figure 4*b*), in line with the data, but then declines, in line with the predictions of Stone *et al*. (2007). More directly in line with the data, epidemic peaks tended to occur later when the rate of increase of susceptible hosts over the breeding season was greater (figure 4*c*; linear regression *R*^2^=0.138, *p*<0.001), and also when abundance at the end of the previous breeding season was lower, although this negative relationship was relatively weak (figure 4*d*; linear regression *R*^2^=0.045, *p*=0.03).

## 4. Discussion

The focal patterns identified by Stone *et al*. (2007) for human infections, where a large epidemic peak in 1 year delayed the onset of an epidemic peak in a subsequent year, were not repeated here. This is perhaps not surprising, given that there is an especially strong contrast between the key characteristics of human populations—(approximately) constant abundance and long lifespan (and hence low population turnover)—and populations of wild rodents. What patterns may be found in larger, longer lived wildlife species remains an open question. Our model results indicate, moreover, that we should not necessarily expect these relationships to be monotonic, and they clearly show that fitting linear regressions to such relationships may grossly misrepresent the true mechanistic relationship.

Stone *et al*. (2007) were able to attribute their small (late) peaks to a shortage of newly recruited susceptible hosts. Following this, a winter with continued recruitment but little disease transmission provided a ready supply of susceptible hosts in the following summer, and consequently a large (early) peak. An association of the timing of epidemics with the supply of susceptible hosts was also found in the present study, but the pattern was not the same. The contrast is between a human population, in which abundance is effectively constant and disease transmission is often strongly seasonal but recruitment is not, and wildlife populations such as ours in which abundance is variable and both recruitment and transmission are seasonal. Thus, in human populations, the supply of susceptible hosts at the beginning of the summer is a straightforward reflection of the extent to which disease reduced the number of susceptible hosts in the previous summer. But in our vole populations, the supply of susceptible hosts over the summer is usually dominated by host rather than infection dynamics. A plentiful supply of susceptible hosts throughout the breeding season (March–September) gives rise to a steadily increasing number of infected hosts and a late peak. A meagre supply may be insufficient to sustain an increase in the numbers infected, leading to a curtailed epidemic, with an early peak followed by a decline.

In fact, the timing of the epidemic peak in our system appears to be associated with both the number of susceptible hosts and their rate of recruitment measured over the whole breeding season. Peaks tended to occur later when there were more susceptible hosts. This is likely to have occurred because the basic reproduction number, *R*_0_ (the average number of secondary infections generated by a primary infection over its infectious lifetime), which increases with the number of susceptible hosts, was relatively large throughout much or all of the summer, and so the numbers infected could continue to increase. But peaks also tended to occur later when recruitment of susceptible hosts was sustained throughout the summer, rather than peaking early itself.

Thus, peaks were most consistently late in 2005 (all in month 11), when recruitment of susceptible hosts was maintained throughout the summer, but the proportion of susceptible hosts also started at a high level as a result of the small epidemics in the previous (crash) year (reminiscent of the patterns found by Stone *et al*. 2007). Peaks were late, too, in other increase-phase years (2002 and 2006), when the number of susceptible hosts was relatively high and their recruitment rate was sustained. However, in the peak year (2003), although the numbers susceptible reached high levels, these peaked early (and then started to crash) and the epidemic peaks were themselves relatively early (one month 7 and three month 9).

Finally, peaks tended to occur earlier in the crash phase (2004), where both the number and the rate of recruitment of susceptible hosts were low, and *R*_0_ was therefore likely to be much closer to, and eventually less than, unity. However, the crash year was also inevitably the most affected by both process and sampling uncertainty, and the variable timing of the epidemic peaks (months 3–9) is likely to have reflected that. In particular, there was a tendency at all sites for ‘twin’ epidemic peaks (figure 1), with the mid-summer decline likely to reflect the shortage of susceptible hosts. In some cases there was no substantive recovery in the infection dynamics following this and the epidemic peaked early, but in others the recruitment of susceptible hosts was eventually sufficient to generate a second, higher and therefore later peak.

The predictions from the model of Smith *et al*. (2008) can in the first place be contrasted with those reported by Stone *et al*. (2007), reflecting the contrasting assumptions they incorporate. Seasonal infection and recruitment can clearly lead to different effects on the timing of seasonal epidemics, and the interplay between them may be even more complicated. Further theoretical studies will be required to look at this interplay. On the other hand, the similarities between the predictions from Smith *et al*. (2008) and the field vole-cowpox data (i.e. between one set of data from one field system and a model aimed at capturing the dynamics of such systems generally) suggest that the empirical patterns reported here have a relevance that extends beyond the system that generated them.

The work of Stone *et al*. (2007) aside, most of the relatively few empirical studies of seasonality in infectious disease dynamics have described, or sought to understand, consistent unvarying patterns (Altizer *et al*. 2006), or, if the patterns have varied, the variation has been linked clearly to external forcing. Thus, Pascual *et al*. (2002) describe regional switches in India from one to two peaks of cholera incidence each year, linked to patterns of precipitation; while Altizer *et al*. (2004) describe a latitudinal cline in the eastern USA in the timing and severity of outbreaks of *Mycoplasma gallisepticum* infection in house finches, linked to milder southern climates. Here, by contrast, similar to Stone *et al*. (2007), we have described variations in timing and severity that are apparently generated within the system itself.

Altizer *et al*. (2006) also identified a series of five ‘future challenges’ for studies of seasonality in the dynamics of infectious disease. Of these, two are reinforced by the present study. First, the need they identify, for mathematical models to move beyond simple formulations (such as sine waves) when seasonality is incorporated, is further accentuated by the variations in the timing and severity of epidemics that have been demonstrated here. Similarly, their challenge of moving beyond speculation in identifying the mechanisms through which seasonality affects infection dynamics is especially evident once the added complexity of the patterns described here is acknowledged. Moreover, a third of their challenges are taken up directly by the present study, namely the need to describe patterns of seasonality in time-series data, especially since, as they point out, almost all previous studies have been on humans. However, their call for methods that can handle ‘hidden’ variables such as the numbers susceptible has been obviated in our study by these numbers having been estimated directly.

Hence, this study supports the contention of Altizer *et al*. (2006) that seasonality adds important additional dimensions to infection dynamics that should not be ignored. The mounting evidence that climate change is transforming the timing and length of growing and breeding seasons (Walther *et al*. 2002) further supports this contention. Our study also supports the underlying pattern demonstrated by Stone *et al*. (2007) in which the supply of susceptible hosts is key to understanding the timing of epidemic peaks. However, the results from our study argue that in many wildlife populations, especially where size is dynamic and lifespans are short, the timing of peaks will be related not just to the seasonal dynamics of infection but also to the seasonal dynamics of host abundance. Moreover, since the seasonal dynamics of hosts are likely to vary among systems (few will show 3–4 year cycles, as here) the detailed seasonal dynamics of epidemic peaks are also likely to be system specific, at least in detail.

## Figures and Tables

**Table 1:** The results of ordinal regressions with, as response variable, either the lunar month when the numbers infected peaked (above, models N1–N6) or the lunar month when the prevalence of infection peaked (below, models P1–P6). (The first column shows the explanatory variables included in the model: *S*-recruitment is ln(number susceptible in month 10/number susceptible in month 3), *S*-number is the sum of the number of susceptible hosts present from months 3 to 10, *N*-previous is peak ln(total numbers) during the previous summer. Successive columns show *b*, the coefficient of the explanatory variable in the model, its standard error, the *Χ*^2^ statistic from the likelihood ratio test for the inclusion of the parameter with its associated *p*-value, and the coefficient of determination, *R*^2^. For models with two explanatory variables, there are two likelihood ratio tests, but there was only a single *R*^2^ value*.)

model variable(s)	*b*	s.e.	*Χ*^2^	*p*-value	*R*^2^
*timing of peaks in the numbers infected*					
N1. *S*-recruitment	1.5	0.68	5.27	0.022	0.24
N2. *S*-number	0.19	0.090	5.07	0.024	0.23
N3. *S*-recruitment+*S*-number	1.7	0.74	6.21	0.013	0.45*
	0.22	0.10	6.01	0.014	0.45*
N4. *N*-previous	−2.9	1.0	10.43	0.001	0.43
N5. *N*-previous+*S*-recruitment	−3.4	1.2	11.92	0.0006	0.60*
	1.9	0.78	6.76	0.009	0.60*
N6. *N*-previous+*S*-number	−2.5	1.1	6.09	0.014	0.48*
	0.085	0.10	0.73	0.39	0.48*
*timing of peaks in the prevalence of infection*					
P1. *S*-recruitment	1.4	0.73	3.95	0.047	0.18
P2. *S*-number	0.21	0.087	6.33	0.012	0.28
P3. *S*-recruitment+*S*-number	1.3	0.72	3.65	0.056	0.40*
	0.21	0.089	6.03	0.014	0.40*
P4. *N*-previous	−2.1	0.87	6.55	0.011	0.29
P5. *N*-previous+*S*-recruitment	−2.2	0.92	6.48	0.011	0.42*
	1.4	0.72	4.12	0.040	0.42*
P6. *N*-previous+*S*-number	−1.5	0.94	2.72	0.10	0.37*
	0.14	0.093	2.50	0.11	0.37*
